# Regulation of Immune Cell Functions by Metabolic Reprogramming

**DOI:** 10.1155/2018/8605471

**Published:** 2018-02-13

**Authors:** Jaehong Kim

**Affiliations:** ^1^Department of Biochemistry, School of Medicine, Gachon University, Incheon 21999, Republic of Korea; ^2^Department of Health Sciences and Technology, Gachon Advanced Institute for Health Science and Technology, Gachon University, Incheon 21999, Republic of Korea

## Abstract

Recent findings show that the metabolic status of immune cells can determine immune responses. Metabolic reprogramming between aerobic glycolysis and oxidative phosphorylation, previously speculated as exclusively observable in cancer cells, exists in various types of immune and stromal cells in many different pathological conditions other than cancer. The microenvironments of cancer, obese adipose, and wound-repairing tissues share common features of inflammatory reactions. In addition, the metabolic changes in macrophages and T cells are now regarded as crucial for the functional plasticity of the immune cells and responsible for the progression and regression of many pathological processes, notably cancer. It is possible that metabolic changes in the microenvironment induced by other cellular components are responsible for the functional plasticity of immune cells. This review explores the molecular mechanisms responsible for metabolic reprogramming in macrophages and T cells and also provides a summary of recent updates with regard to the functional modulation of the immune cells by metabolic changes in the microenvironment, notably the tumor microenvironment.

## 1. Introduction

Pleiotropic interactions between various cells are responsible for the maintenance and disturbance of homeostasis in the tissue microenvironment of physiological and pathological conditions. For example, from early carcinogenesis to progression and metastasis, cancer cells interact with various types of stromal cells, for example, cancer-associated fibroblasts, endothelial cells, and immune cells in the tumor microenvironment (TME). The TME is flooded with cytokines and growth factors responsible for “smoldering persistent inflammation.” This reactive stroma is a well-characterized component of the TME that shows similarities to the repair response in injured tissue [1]. Recent findings revealed that various immune cell subsets are dominant regulators of the delicate balance between homeostasis and disturbance in the tissue microenvironment [2–5]. For example, macrophages can form a major component of immune cell infiltrate in the TME, constituting as much as half of a tumor mass [6, 7]. Immune responses of M1 and M2 macrophages describe the opposing activities of killing or repairing. The typical M1 macrophages drive inflammation and show high antigen presentation, high production of inflammatory cytokines such as IL-12 and IL-23, and high production of nitric oxide (NO) and reactive oxygen intermediates. In contrast, M2-type responses are the “resting” phenotype and are observed in the resolution of inflammation without infections, tissue remodeling, and repair. It has been widely accepted that IFN*γ* alone or with microbial LPS or cytokines such as GM-CSF and TNF induces classically activated M1 macrophages, and IL-4, IL-6, IL-10, IL-13, IL-21, IL-33, immune complexes, and Notch can induce the M2 form of macrophage activation [8, 9]. Notably, truly polarized macrophages are rare [10–13] and tumor-associated macrophages (TAMs) can be also described as M(IL-4), M(Ig), M(IL-10), M(GC: glucocorticoid), M(IFN*γ*), M(LPS), and so forth, according to a recently attempted nomenclature based on specific activation standard [12]. Evidence supports a tumor-promoting role of TAMs, and high frequencies of TAMs are generally associated with poor prognosis in most human cancers [2, 14, 15]. TAMs infiltrating established tumors generally show the properties of an M2-like activated anti-inflammatory, protumoral properties rather than M1-like activated proinflammatory, antitumoral phagocytic properties [16–18].

Macrophages can also form a major component of immune cell infiltrate in obese adipose tissue (AT), constituting as much as 40% of all AT cells [19]. In the progression of obesity, a switch from M2-like to M1-like activation of the macrophage population occurs and inflammatory cytokines such as tumor necrosis factor (TNF) contribute to insulin resistance in adipocytes characterized by an impaired insulin response such as hypertriglyceridemia and elevated fasting glucose [20, 21]. In addition, lymphoid as well as myeloid cells infiltrates and expands in the liver tissue and the obese AT and these immune cell subsets are responsible for the development of obesity-related metabolic dysregulation due to excessive nutrient intake and exacerbation of low-grade inflammatory changes in the microenvironment. CD8^+^ T cells also promote inflammation and metabolic disturbance in the AT [22]. In addition to macrophages and T cells, neutrophils and mast cells can also disturb the homeostasis in the tissue microenvironment.

Many recent findings in the field of immunometabolism now show that metabolic status in immune cells can determine various types of immune responses. Immune cells have remarkably diverse functions and cellular activities that are associated with distinct metabolic demands. The traditional simple concept of production of cellular ATP is that glycolysis generates two molecules of ATPs from one molecule of glucose. Glycolysis metabolizes glucose to pyruvate first, and the pyruvate is further metabolized to carbon dioxide, NADH, and FADH_2_ in the mitochondria. The reducing equivalents (NADH and FADH_2_) drive oxidative phosphorylation (OXPHOS) for more ATP synthesis. In the 1920s, it was demonstrated that cancer tissues can metabolize, even in aerobic conditions, about tenfolds more glucose to produce lactate than normal tissues can and this is known as aerobic glycolysis or the Warburg effect [23]. Since pyruvate is metabolized to lactate and secrete, lactate appears to be wasted in aerobic glycolysis. However, lactate secretion out of cells allows increased continuous glucose influx from the generation of NAD^+^ and resultant accumulation of glycolytic intermediates facilitates biomass synthesis for rapidly proliferating cells. Since the observation and dramatic revitalization of the Warburg effect, the dominant glycolysis and relatively reduced OXPHOS were thought to be confined to cancer cells. However, recent findings clearly show that the Warburg effect-like metabolic reprogramming also exists in rapidly proliferating cells including various types of immune cells, most notably in macrophages and T cells, and determines the function of the immune cell subsets in disease conditions such as those in inflamed tissue or cancer [24–27].

## 2. Metabolic Regulation of Macrophage Phenotypes

The function of macrophages is not limited to the maintenance of homeostasis in the tissue microenvironment but also includes many activities such as cytokine production and phagocytosis upon their activation. Importantly, macrophages are famous for their plasticity and adoption of various activation states in response to their functional requirements signaled from their microenvironment. For example, an innate arm of the immune system can have an important capacity to adapt after challenged with pathogens [28]. This is known as innate immune memory or trained immunity. Trained immunity from epigenetic reprogramming of macrophages shows high glucose consumption and a high ratio of NAD^+^ to its reduced form NADH, reflecting a shift in metabolism with an increase in glycolysis and M1-like activation of macrophages, dependent on the activation of mTOR through the Akt-HIF-1*α* pathway [29]. M2-like activated macrophages exploit fatty acid oxidation (FAO) to fuel OXPHOS rather than aerobic glycolysis for ATP production [30–32].

**Figure 1:**
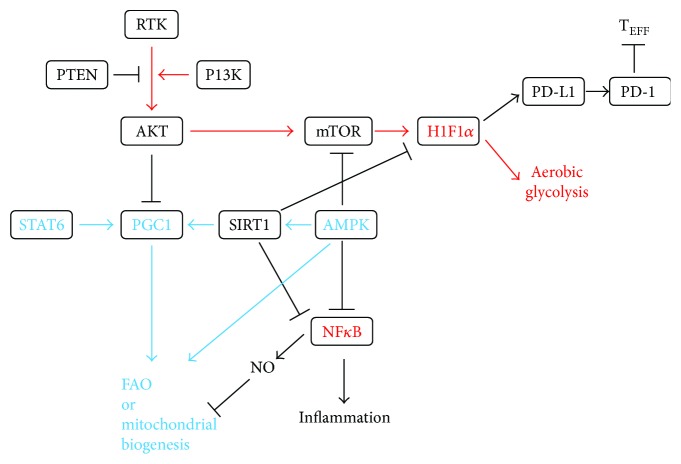
Regulation of metabolic rewiring in macrophages. PGC1 is important for FAO and mitochondrial biogenesis (shown in blue) and HIF1*α* for aerobic glycolysis (shown in red). Active SIRT1 can inhibit inflammation and glycolytic metabolism and promote mitochondrial biogenesis and FAO. Interaction of PD-L1 and PD-1 induces FAO and suppresses aerobic glycolysis and immune functions in Teff. PD-1: programmed death-1; PD-L1: programmed death ligand-1; RTK: receptor tyrosine kinase; T_EFF_: effector T cell.

**Figure 2:**
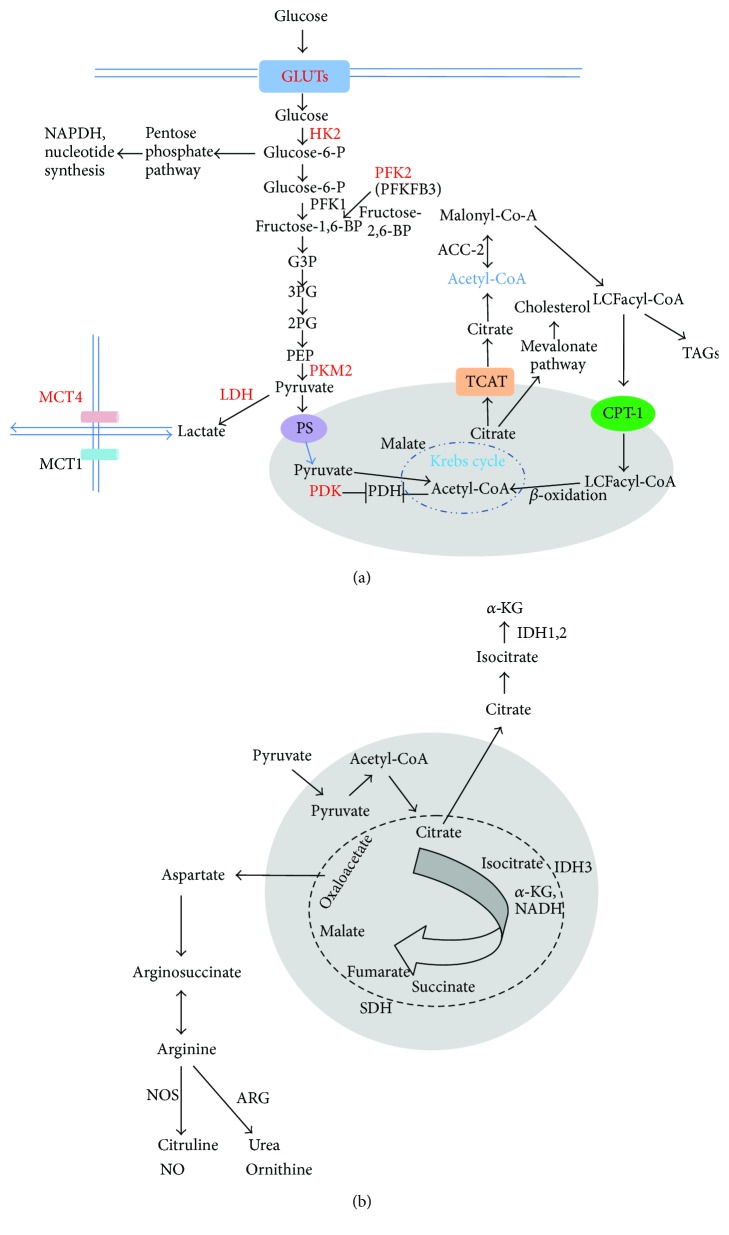
Metabolism of glucose and fatty acid at a glance. (a) Stabilization of HIF-1*α* upregulates GLUTs, HK2, PFK2, PKM2, LDH, PDK, and MCT4 shown in red. ACC: acetyl-CoA carboxylase; ARG: arginase; CPT-1: carnitine palmitoyltransferase 1; FAT: fatty acid translocase; G3P: glyceraldehyde 3-phosphate; GLUT: glucose transporter; HK2: hexokinase 2; IDH: isocitrate dehydrogenase; LCFacyl-CoAs: long-chain fatty acyl-CoAs; MCT: monocarboxylate transporter; 2PG: 2-phosphoglycerate; 3PG: 3-phosphoglycerate; PEP: phosphoenolpyruvate; PDH: pyruvate dehydrogenase; PDK: pyruvate dehydrogenase kinase; PFK: phosphofructokinase; PS: pyruvate symporter; SDH: succinate dehydrogenase; TAG: triacylglyceride; TCAT: tricarboxylic acid transporter. (b) A schematic of the Krebs cycle and metabolites exported out of the mitochondria. Arginine is metabolized to urea and ornithine in M2-like macrophages that do not express NOS. ARG: arginase; NOS: nitric oxide synthase.

Of note, HIF1*α* and NF*κ*B drive the M1 phenotypes [33, 34] and PGC1*β*, and peroxisome proliferator-activated receptors and STAT6 drive the M2 phenotypes (Figure 1) [35–38]. Phosphorylation and activation of a nutritional sensor, AMPK, regulate mitochondrial biogenesis via deacetylation of regulating proteins, including SIRT1 with NAD^+^, and suppress HIF1*α* and NF*κ*B [38–40]. AMPK and NAD^+^-SIRT1-PGC1*β* signaling are key factors for nutritional state-dependent M1/M2-like activation of macrophages in inflammatory conditions [39, 41]. HIF-1*α* also enhances the lactate dehydrogenase- (LDH-) mediated conversion of pyruvate-to-lactate [42] and increases expression of GLUT1, GLUT3, and MCT4 to increase glucose uptake and expression of pyruvate kinase M2 (PKM2), resulting in an increase in the secretion of lactate and uncoupled glycolysis and oxidative phosphorylation [43, 44] (Figure 2). Pyruvate dehydrogenase (PDH) inactivation from phosphorylation by pyruvate dehydrogenase kinases (PDKs) prevents pyruvate from entering the mitochondrial Krebs cycle [45]. HIF-1*α* transcriptionally activates the PDKs [46, 47].

LPS-activated dendritic cells and M1-like activated macrophages show enhanced aerobic glycolysis, flux through the pentose phosphate pathway, and fatty acid synthesis but have incomplete OXPHOS at the level of succinate dehydrogenase (SDH) and isocitrate dehydrogenase, blocking the synthesis of mitochondrial ATP. In these cells, glucose is used for the biosynthesis of large quantities of cytokines and effector molecules, and inactivation of OXPHOS directs metabolites from the Krebs cycle for inflammatory reaction [32, 48]. Accumulation of succinate and citrate from the truncated OXPHOS leads to stabilization of HIF1*α* by limiting prolyl hydroxylase activity to maintain a proinflammatory, antitumoral response [49–51].

Recently, itaconic acid-mediated inhibition of SDH has also been found as a driver for succinate accumulation in LPS-stimulated M1-like activated proinflammatory macrophages [52]. Immunoresponsive gene 1 (*Irg1*) is highly expressed in mammalian macrophages during inflammation and *Irg1* gene silencing in macrophages results in significantly decreased intracellular itaconic acid levels as well as significantly reduced antimicrobial activity during bacterial infections [53].

High intracellular iron levels in M1-like activated macrophages stabilize HIF1*α* through low levels of ferroportin and high levels of H-ferritin, involved in iron export and storage, respectively [54, 55]. Hemeoxygenase-1 (HO-1) catabolizes heme to ferrous ion, biliverdin, and carbon monooxide, and suppression of HO-1 results in M2-like activation of TAMs [56].

**Figure 3:**
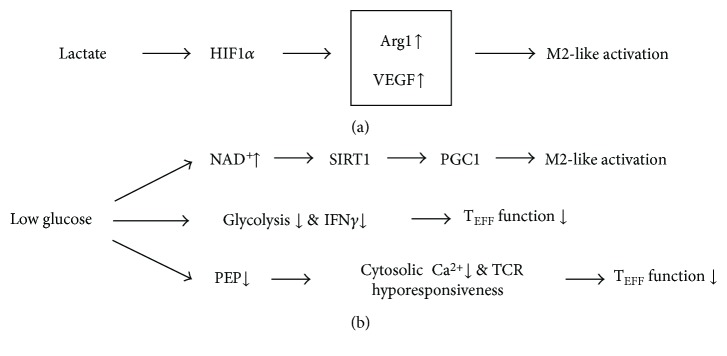
Metabolic changes in the TME-regulating immune cell function. (a) Lactate produced by cancer cells, as a by-product of aerobic glycolysis, has a critical function in inducing M2-like activation of TAMs. (b) A low-glucose microenvironment via multiple signaling pathways regulates activation state of macrophages and T cells. TCR: T cell receptor; VEGF: vascular endothelial growth factor.

HIF1*α* can also be stabilized from nitrosylation with peroxynitrites from increased iNOS [57], favoring aerobic glycolysis in M1 phenotypes. NF*κ*B transcriptionally activates proinflammatory genes including iNOS, which forms NO in the presence of arginine. Peroxynitrite, formed from NO and superoxide anions in the mitochondria, nitrosylates iron-sulfur proteins in the mitochondrial electron transport chain, and the resultant nitrosylation can inhibit OXPHOS [58], also favoring aerobic glycolysis in M1 phenotypes. Unlike iNOS-mediated catabolism of arginine to NO in M1-like activated macrophages, M2-like activated macrophages catalyze arginine to urea and ornithine by arginase 1 (ARG1); ARG1 is a representative marker for M2-like activation. As NO production is limited in M2-like activated macrophages, the nitrosylation-mediated inhibition of OXPHOS is dampened, now favoring M2 phenotypes [48]. Although HIF1*α* drives the M1 phenotypes in hypoxic conditions, lactate produced by cancer cells, as a by-product of aerobic glycolysis, has an unexpected critical function in HIF1*α*-dependent expression of ARG1 and resultant M2-like activation of TAMs in normoxic conditions [59] (Figure 3). These findings clearly indicate highly interconnected signaling for the conservation of HIF-1-centered metabolic phenotypes.

As stated, M2-like activated macrophages show lowered glycolysis and enhanced FAO to fuel OXPHOS. Th2 cytokine and IL-4-induced PGC1*β* increase mitochondrial biogenesis and FAO in a STAT6-dependent manner [38, 41, 60]. PGC1*β* plays a key role in increasing mitochondrial biogenesis and OXPHOS by upregulating the expression of FAO-involved genes [41]. IL-4-/IL-13-stimulated macrophages express PFKFB1, which produces a low level of a glycolytic activator, fructose 2,6 bisphosphate [61, 62]. In IL-4-stimulated macrophages, fatty acid sources such as LDL and VLDL are taken up via the scavenger receptor CD36 and metabolized in the lysosome. The CD36-mediated lysosomal lipolysis is essential for the M2-like activation [31].

An orphan nuclear receptor, estrogen-related receptor *α* (ESRR*α*), is required for the increased mitochondrial biogenesis [63]. Importantly, ESRR*α*-deficient macrophages show a decrease in phagosomal maturation and antimicrobial activity [64]. Another study reported an M1-like phenotype of increased glycolysis but impaired mitochondrial respiratory function and biosynthesis as a result of ESRR*α* deficiency [65]. Interestingly, VLDLR expression is a determinant factor in inflammation and in M1-like activation of macrophages in AT [66].

In spite of our knowledge gained from macrophages in inflammatory disease conditions, our understanding of the metabolic regulations in TAMs is surprisingly limited and the signals involved in communication between tumors and macrophages are still poorly defined [67]. However, emerging evidence strongly indicates that the metabolic reprogramming of macrophages is closely related to the protumoral or antitumoral function of macrophages [68, 69] and that unraveling the TAM phenotype might lead to the identification of alternative, novel metabolic targets for TAM-directed intervention. Recently, it was shown that lactate produced by cancer cells has a critical function in inducing M2-like activation of TAMs [59] (Figure 3). Acidification of the TME by lactate increases level of ARG1, a representative M2 marker, in macrophages, which limits the proinflammatory, antitumoral response of TAMs and, importantly, the proliferation and activation of T cells [59, 70]. Also, de novo fatty acid synthesis in cancer cells increases fatty acid levels in the TME to promote the generation of immunosuppressive, regulatory T cells (Tregs) and M2-like TAMs, favoring survival of cancer cells [71]. Expression level of vitamin D receptor (VDR) negatively correlates with metastasis in breast cancer, and suppression of VDR by TNF*α* can mediate the prometastatic effects of TAMs through enhancement of the *β*-catenin pathway [72].

## 3. Metabolic Regulation of T Cells

Multiple studies have shown that distinct metabolic programs in CD4^+^ T cell subsets can be manipulated *in vivo* to control Treg and effector T cells (T_EFF_) development in inflammatory diseases [73–76]. A transcription factor, Myc, shows a dominant role in driving metabolic reprogramming in activated T cells by promoting glycolysis and glutaminolysis and suppressing FAO [75]. mTOR increases expression of HIF-1*α*, which facilitates the expression of critical glycolytic enzymes and promotes differentiation and activation of T cells [76].

A “shift” from OXPHOS to aerobic glycolysis is a hallmark of T cell activation [25]. T cells, if not activated, show low levels of metabolic requirements, use OXPHOS to maximize production of ATP as an energy source, and engage scarcely in biosynthesis, while activated T cells use aerobic glycolysis to produce effector molecules for rapid cellular proliferation [32].

In order to facilitate proper immunological response upon encounter of antigenic stimuli, it is vital that T cells should differentiate into T_EFF_ and clonally expand rapidly to ensure prompt reaction. Glycolysis promotes the differentiation of activated CD4^+^ T cells into T_EFF_ [73]. Activated T cells also consume glutamine to fuel the Krebs cycle to support the production of biomass and ATP [77]. Clonal expansion is achieved from upregulation of glycolysis and OXPHOS together. In addition to high level of glycolysis, increased mitochondrial flux and production of ROS are also required for initiation of the clonal expansion [78]. After differentiation, T_EFF_ cells, Th1, Th2, and Th17 cells, remain highly glycolytic [73].

When the antigenic stimuli are eliminated, most T_EFF_ cells die, leaving behind a small antigen-specific T cell population that becomes memory T cells (Tm). Quiescent Tm with the CD8 coreceptor exploits FAO to fuel OXPHOS rather than aerobic glycolysis for ATP production [32, 73]. Instead of utilizing extracellular lipids for energy generation, Tm metabolizes de novo generated fatty acids, synthesized from extracellular glucose and intracellularly stored during the previous effector phase [79]. Enforcing FAO with activation of AMPK or inhibiting mTOR results in increased numbers of Tm [80–82]. Mitochondrial oxidative metabolism supports immunosuppression and lineage commitment of Tregs [83–85]. Tregs with increased glycolysis are more proliferative yet have reduced ability to maintain FOXP3 expression and suppress inflammation [84].

## 4. Nonmetabolic Function of Glycolytic Enzymes in Immune Cells

In addition to their canonical, metabolic functions in glycolysis, recent studies uncovered nonmetabolic functions of glycolytic enzymes such as hexokinase 2 (HK2), phosphoglucose isomerase, and GAPDH, connecting metabolic states to apoptosis, gene transcription, protein kinase activity, and the mTOR signaling pathway [86]. Briefly, the interaction between HK2 and voltage-dependent anion channel (VDAC1) reduces the release of proapoptotic proteins and prevents cancer cells from undergoing apoptosis [87]; phosphoglucose isomerase exerts its antiapoptosis effect by suppressing the expression of Apaf-1 and caspase-9 genes, thereby indirectly regulating the formation of the apoptosome [88, 89]. GAPDH exerts controversial pro- and antiapoptotic effects through interaction with VDAC1 and induction of autophagy, respectively [86, 90].

Other studies have shown that GAPDH, HK, and enolase are also RNA-binding proteins [91–93] and the “REM (RNA–enzyme–metabolite) hypothesis” proposes a regulatory interaction between gene expression and cellular metabolism by RNA-binding metabolic enzymes [94, 95]. It is notable that many glycolytic enzymes, formerly known to exclusively function in glycolytic metabolic events in the cytoplasm or mitochondria, have now been shown to regulate transcription and translation [86, 91]. Indeed, numerous metabolic enzymes that also function in glycolysis, fatty acid synthesis, and the Krebs cycle are also RNA-binding proteins [96], although the significance for immune response is not clearly known except for that of GAPDH [95] and enolase [32, 83]. Recently, a REM connection with GAPDH was proven in T cell activation [25]. GAPDH is diverted to glycolysis and translation of IFN*γ* and IL-2 is not perturbed in highly glycolytic T cells. However, when aerobic glycolysis is blocked, GAPDH binds to IFN*γ* and IL-2 mRNA in CD4^+^ T cells to suppress their translation [25]. In myeloid cells, GAPDH is a component of the IFN*γ*-activated inhibitor of translation (GAIT) complex that controls translation of inflammatory genes [97, 98]. High glycolytic flux suppresses the interaction between GAPDH and Rheb and thus allows Rheb to activate mTORC1 and stimulate cell growth [99]. By modulating expression of Foxp3-splicing variants with exon 2(Foxp3-E2), enolase-1-mediated glycolysis controls induction of human Tregs with a potent immunosuppressive function [83]. When glycolysis is inhibited, enolase-1 translocates to the nucleus and represses expression of the Foxp3-E2 splice variant in Tregs and suppresses Treg induction.

The level of the glycolytic intermediate phosphoenolpyruvate (PEP) is controlled by a balance between enolase-mediated formation of PEP and pyruvate kinase-mediated conversion to pyruvate. PKM2 exists either as an inactive dimer or as more active tetramer, and the transition between the two conformations is subject to posttranslational modifications [100]. Dimeric PKM2, previously regarded as crucial for metabolic reprogramming exclusively in cancer cell, is also important in promoting aerobic glycolysis in immune cells [101, 102]. Enhanced expression of dimeric PKM2 reduces the rate of PEP conversion to pyruvate and results in an accumulation of glycolytic products that can be otherwise metabolized in biosynthetic pathways [32]. Importantly, PEP enhances antitumor effector functions in activated T cells by regulating Ca2^+^ import into the endoplasmic reticulum, thus sustaining translocation of nuclear factor of activated T cells (NFAT) into the nucleus and the expression of a set of genes that are required for T cell activation [103].

These findings imply that a direct and strong interaction exists between the nonmetabolic function of glycolytic enzymes and the generation of immune responses and also that enhanced glycolysis sustains antitumoral and proinflammatory functions via highly interconnected signaling in immune cells.

## 5. Metabolic Changes in the TME Influencing Immune Cell Functions

The microenvironment determines the metabolism of immune cells, which in turn adjust to a broad spectrum of configurations to meet the demands of various cellular activities. For example, changes in the metabolic profiles of immune cells by cancer cells can alter the function of the immune cells [103, 104]. A protracted aerobic glycolysis acidifies and destabilizes the TME and this is consistent with the view of the tumor as an unhealed wound [105]. Similarities of utilizing nutrients and engaging metabolic regulation to sustain cellular proliferation and survival are shared by cancer and immune cells. Notably, nutritional competition between cancer cells and antitumoral immune cells in the TME shifts the activation and differentiation status of T-cells to favoring the survival of cancer cells [103, 104, 106, 107].

Recently, it was shown that lactate produced by cancer cells, as a by-product of aerobic glycolysis, has a critical function in signaling that induces M2-like activation of TAMs [59] (Figure 3). Interestingly, lactate-induced M2-like activation was from HIF1*α*-dependent expression of ARG1. Depletion of glucose and a glucose-rich hypoxic ROS environment favor M2-like activation and M1-like activation of TAMs, respectively, and depletion of glucose can disarm T cells in the TME [27, 104]. Low levels of ATP from dietary restrictions or energy consumption induces nicotinamide phosphoribosyltransferase that generates NAD^+^, which is a key factor for SIRT1 activation. SIRT1 acetylates and activates PGC1*β* to increase OXPHOS [35, 67, 108]. Pyruvate is metabolized by LDH-A, producing lactate and NAD^+^. NAD^+^ acts as an electron acceptor in the Krebs cycle and the electron transport system in mitochondria. It appears feasible from these findings that glucose-depleted, low ATP, and NAD^+^-rich states (in cachexic patient with advanced cancer) may drive the M2-like activation of macrophages, while the macrophage population still retains its phagocytic activity in maintaining biosynthesis with molecules acquired from their microenvironment [32]. For the identification of alternative, novel targets for TAM-directed intervention, it would be necessary to show whether these events can predominantly happen in TAMs of the TME.

A recent study observed that hypoxia-induced upregulation of the immunosuppressive programmed death ligand-1(PD-L1) is directly mediated by HIF1*α* [109]. In the TME, cancer cells, macrophages, and dendritic cells express PD-L1, a notable ligand for immune checkpoint, programmed cell death-1(PD-1) in T_EFF_. The interaction of PD-1 and PD-L1 directly inhibits glycolysis and promotes lipolysis and FAO in T cells, resulting in failure of the antitumoral function of T cells [110] (Figure 1). The lessons that application of immune checkpoint blockade antibodies against cytotoxic T lymphocyte antigen-4 (CTLA-4), PD-1, and PD-L1, which are used clinically, restore glucose in the TME, permitting T cell glycolysis and IFN*γ* production clearly show that nutrient availability in the microenvironment can change the metabolic status of immune cells. Another study also revealed that PD-1 expression by TAMs correlates with protumoral activity, and blockage of PD-1–PD-L1 *in vivo* increases phagocytosis, reduces growth of cancer cells, and increases the survival of mice in mouse models of cancer in a macrophage-dependent way [111]. In addition, blocking PD-L1 directly on cancer cells decreases glycolysis and restores glucose in the TME, resulting in allowance of antitumoral function of T cells from glycolysis and IFN*γ* production [104].

In conclusion, these findings indicate that signals such as cytokines, growth factors, hypoxia, and nutrient availability that emanate from the microenvironment can induce metabolic changes in immune cell subsets, resulting in changes in immune functions and pathological responses. An interesting perspective is whether immune cells can supply their microenvironment with lactate and antioxidative resources as stromal cells in the TME are able to (the reverse Warburg effect). Considering their preponderance in the TME, there is an ample possibility that metabolic changes in a large subgroup of macrophages or T cells may also affect the metabolic state of their microenvironment and functions of other cellular components.

## 6. Potential Metabolic Targets for the Manipulation of Immune Cell Function

Since the function of immune cells is dependent on a delicate metabolic balance, results of many clinical trials performed with inhibitors of metabolic enzymes and oncogenes will provide valuable insights for the prospect of immunomodulation by specific metabolic regulation [112]. Of note, results from targeting cancer metabolism *in vivo* have been disappointing and less prominent than results from targeting immune cell metabolism [85]. The PKM2 inhibitor, TLN-232, was tested in a clinical trial for refractory renal cell carcinoma (NCT00422786). Inactive dimeric PKM2 activates the mTORC1 signaling pathway by phosphorylating the mTOR inhibitor, AKT1S1, and leads to an accelerated oncogenic growth and autophagy inhibition of cancer cells [113]. In line with this, increase in the tetrameric, active form of PKM2, attenuated the LPS-induced proinflammatory M1-like macrophage phenotypes while promoting M2-like macrophage phenotypes [114]. Many AMPK activators are now tested in clinical and preclinical studies for diabetes, cancer, and cardiovascular disease [115]. Importantly, AMPK stimulation inhibiting mTORC1 was sufficient to decrease Glut1 and increase generation of Tregs in an animal model, implying AMPK activation as a potential manipulable checkpoint for immune response [73]. REDD1, an inhibitor of mTOR, is highly expressed in M2-like TAMs. Inhibition of REDD1 stimulates glycolysis in the TAMs and competition of glucose between TAMs and endothelial cells prevents vascular hyperactivation and promotes the formation of quiescent vascular junctions in the TME [69]. Suppression of REDD1 was attempted in phase 2 clinical trial (NCT00713518) for the treatment of neovascularization in AMD patients. Nitrosylation of HIF1*α* prevents its degradation. If denitrosylation of HIF1*α* is observed, its modulation may be potentially applicable for the inhibition of glycolytic enzymes and the alleviation of M1-like phenotypes.

Isoprenylation of ubiquinone is important for OXPHOS and isoprenylation of Ras, Rho, and Rab guanosine triphosphatases is involved in immunological synapse formation, migration, proliferation, and cytotoxic effector response of T cells. The intracellular availability of sterols is crucial for isoprenylation modification of proteins for plasma membrane attachment and represents a checkpoint for metabolic reprogramming that modulates T cell responses [116]. Statin and other chemical inhibitors of the mevalonate pathway can suppress isoprenylation of Rho proteins [117] and have been tested in many clinical trials.

Clinical trials involving agents that inhibit PD-L1 and PD-1 are now being performed. Atezolizumab is the sole member of this class currently approved for the treatment of bladder cancer, but approvals for avelumab, durvalumab, nivolumab, and pembrolizumab in the treatment of various cancer are anticipated in the near future [118]. Therefore, it appears possible that the combined use of metabolism-targeting reagents with immune checkpoint inhibitors can alter the activation and differentiation of T cells.

## 7. Conclusions

Immunity and metabolism advance together. Considering the significant contribution of immune cell functions in promoting and suppressing various types of disease progression, repolarization of immune cells from the potential targets stated above shows an ample possibility to become novel therapeutic approaches. Extension of our knowledge of the functional plasticity of macrophages and T cells spanning from inflammation biology to cancer immunology and the persistent reprogramming effect achievable from stable epigenetic changes in the metabolic pathways of macrophages [29] and potentially T cells by potential modulators may provide new information for immune therapeutic strategies applicable for different disease conditions. Importantly, cancer cells and host primary cell constituents such as immune cells and stromal cells can form microanatomical compartments within the cancer tissue to regulate metabolic needs, immune surveillance, survival, invasion, and metastasis. Indeed, different signals from particular locations in the TME seem to influence activation of TAMs and T cells and overall tumor prognosis [119]. TAMs can be diverse within the microanatomical compartments, including the accumulation of M1-like activated cells with protumoral properties in hypoxic areas [120] and differences in inflammatory components and pathways between tumors originating in distinct anatomical sites [120, 121]. The notion that metabolic competition between cancer cells, immune cells, and other stromal cells can determine function and fate of each cell subset proposing that identification of which of specific niches in the microenvironment can impede immune cells from proper metabolic engagement will encourage significant contributions to this research field. Generation of metabolically fit T cells prior to adoptive cell transfer will improve T cell-based immunotherapy against cancer by surviving the unfavorable, hostile TME. Furthermore, successful therapies targeting the function of macrophages and T cells will require identification of targets that specifically allow metabolic reprogramming of immune cells while, at the same time, not causing an increase in proliferation and survival of cancer cells or systemic inflammatory changes or autoimmunity. Our understanding of the metabolic regulations in B cells is surprisingly limited, and the mechanisms about how cellular metabolism supports and regulates function of B cells are still poorly defined. B cell immunometabolism is anticipated to become an exciting research field.
